# Warming waters lead to increased habitat suitability for juvenile bull sharks (*Carcharhinus leucas)*

**DOI:** 10.1038/s41598-024-54573-0

**Published:** 2024-03-14

**Authors:** Lindsay Mullins, John Cartwright, Steven L. Dykstra, Kristine Evans, John Mareska, Philip Matich, Jeffrey D. Plumlee, Eric Sparks, J. Marcus Drymon

**Affiliations:** 1 Coastal Research and Extension Center, Mississippi State University, Biloxi, MS USA; 2 Northern Gulf Institute, Starkville, MS USA; 3 Department of Wildlife, Fisheries, and Aquaculture, Mississippi State University, Mississippi State, MS USA; 4 College of Fisheries and Ocean Science, University of Alaska Fairbanks, Fairbanks, AK USA; 5 Alabama Department of Conservation and Natural Resources, Dauphin Island, AL USA; 6 Saving the Blue, Cooper City, FL USA; 7 School of Renewable Natural Resources, Louisiana State University Agricultural Center, Baton Rouge, LA USA; 8 Mississippi-Alabama Sea Grant Consortium, Ocean Springs, MS USA

**Keywords:** Ecology, Climate-change ecology, Climate-change ecology

## Abstract

Coastal ecosystems are highly vulnerable to the impacts of climate change and other stressors, including urbanization and overfishing. Consequently, distributions of coastal fish have begun to change, particularly in response to increasing temperatures linked to climate change. However, few studies have evaluated how natural and anthropogenic disturbances can alter species distributions in conjunction with geophysical habitat alterations, such as changes to land use and land cover (LU/LC). Here, we examine the spatiotemporal changes in the distribution of juvenile bull sharks (*Carcharhinus leucas*) using a multi-decadal fishery-independent survey of coastal Alabama. Using a boosted regression tree (BRT) modeling framework, we assess the covariance of environmental conditions (sea surface temperature, depth, salinity, dissolved oxygen, riverine discharge, Chl-a) as well as historic changes to LU/LC to the distribution of bull sharks. Species distribution models resultant from BRTs for early (2003–2005) and recent (2018–2020) monitoring periods indicated a mean increase in habitat suitability (i.e., probability of capture) for juvenile bull sharks from 0.028 to 0.082, concomitant with substantial increases in mean annual temperature (0.058°C/yr), Chl-a (2.32 mg/m^3^), and urbanization (increased LU/LC) since 2000. These results align with observed five-fold increases in the relative abundance of juvenile bull sharks across the study period and demonstrate the impacts of changing environmental conditions on their distribution and relative abundance. As climate change persists, coastal communities will continue to change, altering the structure of ecological communities and the success of nearshore fisheries.

## Introduction

Nearshore fish communities are highly vulnerable to the impacts of climate change and human stressors^1^. As such, these systems face a suite of challenges, including habitat loss, overfishing, invasive species, warming, acidification, and eutrophication^2^, which can cause shifts in the distributions of fish searching for habitable conditions^3^. For example, warming oceans have altered the distribution of tiger sharks (*Galeocerdo cuvier*) both spatially and temporally, with poleward shifts and earlier migration timings^4^. Climate change projections into the twenty-first century indicate that these range shifts are not unique to tiger sharks, and suitable thermal habitat for many species on the North American shelf, including elasmobranchs, will change in both area and location^5,6^. As such, characterizing suitable habitat with applications for future projections will be essential for proactive management of vulnerable coastal fish populations.

In coastal habitats, the effects of climate change are particularly acute for species that are already at risk, such as sharks, which have undergone an 18-fold increase in extinction risk since 1980^7^. Approximately 1/3 of chondrichthyan species are threatened by overfishing^8^, with the greatest threats in coastal shelf waters. This risk is exacerbated by their generally conservative life history, which is characterized by later maturity, lower reproductive output and high levels of maternal investment^9^. Despite the grim global status quo, the United States has uniquely demonstrated that successful science-based management of shark fisheries are possible^10,11^. Yet traditional static spatial management efforts to protect shark habitat are likely to be undermined by habitat shifts attributed to climate change^6^, which means managers will need to proactively anticipate these changes and adjust protections accordingly. Warming waters and other environmental changes resulting from climate change have been shown to impact the habitat use of sharks^8,12,13^. For example, as temperatures warm and salinity regimes change, juvenile bull sharks (*Carcharhinus leucas*), a subtropical to tropical coastal species, have expanded their nursery habitat northward, inhabiting more traditionally temperate estuaries^14^. While bull sharks have exhibited natal philopatry, meaning mothers return to the same estuaries in which they were born for parturition, the expansion of nursery habitat in the Atlantic demonstrates plasticity of this life history strategy in response to climate change^15^. Further, it is predicted that changes in LU/LC will exacerbate the impacts of climate change on water quality parameters, such as temperature, total suspended solids, and freshwater flows for the Gulf of Mexico, underscoring the importance of characterizing the habitat at current conditions^16^. As such, identifying the bounds of environmental parameters characterizing suitable bull shark habitat will be essential to guide management decisions, particularly as shifting environmental conditions require dynamic spatial protections for sharks^6^.

Managing predator populations, such as sharks, is important because of the role they play in shaping ecosystem structure through top-down and knock-on effects^17^. Consequently, changes in the abundance, distribution, and/or behavior of sharks can alter ecological communities. For example, sharks impose a fear-driven behavioral response on sea turtles, which mitigates overgrazing of foundationally important seagrass habitat^17^. Conversely, in Bermuda, where shark populations have been greatly reduced, the reduction in predation risk has resulted in significant decline or complete collapse of seagrass meadows where mesopredator grazing has gone unchecked^17^. As such, the loss of sharks in a system incurs a loss of the services provided by such a keystone species. With regard to conservation efforts, distributional shifts can pose a considerable challenge as delineated management areas (e.g., Highly Migratory Species closed areas^18^) previously identified as relevant habitat may no longer render appropriate protection^19^. Given changing conditions, species distribution modeling (SDM) has been employed to identify lapses in appropriate coverage across a changing landscape^18^. When incorporated with real-time observations of a changing climate, SDMs are powerful tools to help ecosystem managers understand where species densities have shifted as they occupy new geographic ranges and thus, identify where protections may need to be adjusted^18^. Further, projected environmental conditions can be input to predict how those ranges may change in the future. However, published data is lacking for many regions and species, which hinders the implementation of this approach.

Juvenile bull sharks function as unique regulatory predators, globally inhabiting tropical and subtropical coastal ecosystems^20^. They are unique because they have the most wide-ranging salinity tolerance of sharks, allowing them to occupy low salinity estuaries as nursery habitat that are intolerable to many other shark species^21–23^. Estuarine residency reduces the threats of predation and competition during a vulnerable life stage^24^, although these fitness benefits have been difficult to quantify among many populations. Within the northeast Gulf of Mexico, Bethea et al. found that when compared to Mississippi and northwest Florida, young-of-year bull sharks uniquely dominated Mobile Bay’s shark assemblage^25^. Indeed, Parsons & Hoffmayer captured juvenile bull sharks in low salinity habitat in Mississippi-Alabama waters, at locations where no other shark species were captured, indicating bull sharks occupy a unique niche exclusive to other shark species in this region^26^. As such, the nearshore habitats of Alabama are important for effective management of bull sharks in the northern Gulf of Mexico.

The Mobile River Basin is a well-documented North American aquatic biodiversity hotspot that terminates at Mobile Bay, a shallow, seasonally stratified, highly dynamic estuary tangential to coastal Alabama^27^. Terrestrial nutrients and freshwater from the Mobile and Tensaw Rivers mix with the oceanic waters of the Gulf of Mexico to provide suitable habitat for a diversity of fish taxa, including large predators^28^. However, both fresh- and salt-waters are susceptible to warming associated with climate change^29,30^. In the northern Gulf of Mexico, water temperatures have risen an average of 0.05 °C/yr over the last four decades^29^. The coastline has also experienced increases in human population density and tourism, which are projected to continue and have resulted in the conversion of natural to urban land cover^31^. These land-use/land-cover (LU/LC) changes are predicted to alter freshwater inflow, temperature, total suspended solids, and salinity in coastal Alabama^16^. Given that coastal Alabama has one of the highest and most intense rainfalls in the contiguous US (136 cm yr^−1^)^32^, the resultant heightened impact of widespread LU/LC conversion on stormwater runoff merits concern regarding water quality. As a riverine-dominated estuarine system, increases in upstream pervious surface coverage can increase nutrient loading from the surrounding drainage, resulting in eutrophication and subsequent low DO events^33^. Further, Mobile Bay has experienced historic declines (57–88% loss) of submerged aquatic vegetation (SAV) since the twentieth century^16^, reducing its capacity for biofiltration of river-derived nutrients, a natural buffer against eutrophication^34,35^. Warming temperatures, increased eutrophication, and decreased biological filtration of nutrients make the coastal Alabama ecosystem especially susceptibility to the impacts of climate change.

The goal of the current study was to use machine learning models and spatial statistics to assess the distribution and relative abundance of juvenile bull sharks in Mobile Bay. Specifically, we used a multi-decadal fishery-independent dataset to assess spatiotemporal changes in distribution and relative abundance, and examined a suite of environmental factors associated with juvenile bull shark habitat suitability across a broad spatiotemporal scale. In accomplishing this, we were able to address how the abundance of juvenile bull sharks and the extent of their suitable habitat has changed in recent decades, as well as quantify the environmental impacts associated with these changes. This work will fill fundamental gaps in our understanding of how juvenile bull sharks alter their habitat use in response to a changing climate.

## Methods

### Study site

Mobile Bay is a shallow, drowned river valley estuary that is highly variable along the transitional river-sea gradient. It is characterized by an extensive delta region at its northern extent, where the Mobile River empties 90% of the freshwater input into the system^36^. Strong seasonal inflow drives salinity trends, with high flow, low salinity periods occurring February-April and low flow, high salinity occurring August-October^36^. River discharge entering the northern bay has a range exceeding two orders of magnitude, making it an extension of the river during peak flows in winter and spring^37^. The bay is moderately stratified year-round, with the strongest gradients occurring in the springtime as a response to increased river discharge^36^. An overall latitudinal salinity gradient is apparent, with fresher waters at the northern portion of Mobile Bay becoming increasingly saline and stable southward, where Gulf of Mexico waters are exchanged at Main Pass^36^. The southern extent of the bay exhibits less thermal range, where it tends to be warmer in the winter and colder in the summer than the northern portion of the upper bay^36^. The Coriolis force and the triangular shoreline configuration deflect Gulf waters along the eastern shore of Mobile Bay, while fresh waters are deflected to the western shore^36^. Overall, thermal stratification is minimal, with less than 1 °C difference between surface and bottom level waters^36^.

### Field work

**Figure 1:**
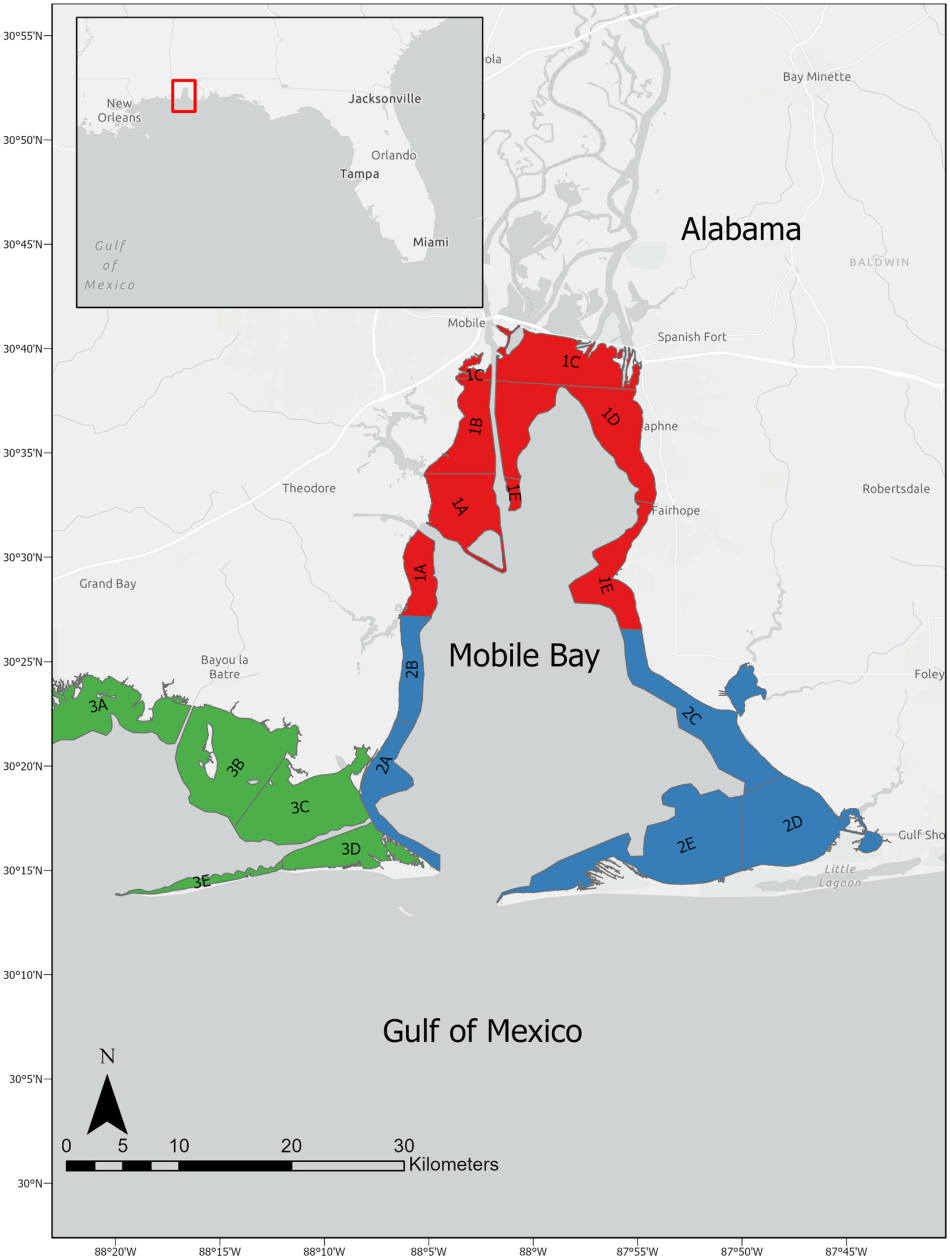
Extent of gillnet survey area along the Alabama coast. Colored areas indicate major sampling areas. Major sampling areas are denoted by number (1–3), and subareas by major area number, then A-E. Inset map denotes extent of sampling coastline in the northern Gulf of Mexico.

Year-round gillnet surveys have been conducted by the Alabama Department of Conservation and Natural Resources-Marine Resources Division (AMRD) since 2001. For this survey, small and large mesh gillnets were used. Small mesh gillnets were divided into five panels (2.44 × 45.7 m), with mesh sizes ranging from 5.1 to 10.2 cm, each panel increasing incrementally by 1.27 cm. Environmental conditions permitting, these nets were set perpendicular to the shoreline with the smallest mesh size closest to shore. Large mesh gillnets were divided into four panels (2.44 × 45.7 m) ranging from 11.4 to 15.2 cm, with each panel increasing incrementally by 1.27 cm. Large mesh nets were set parallel to the shore to a maximum depth of 8 ft. Both net types were soaked for one hour, and were deployed according to a stratified sampling design. The Alabama coastline was divided into three major sampling areas, each of which contained smaller subareas (Fig. 1). Each year, 240 nets were set for sampling, with specific locations within subareas selected at random with boating conditions allowing. Sampling does not necessarily occur at the exact same coordinates as previous occurrences when a subarea is resampled. Sampling efforts were increased in the summertime, when diversity of catches and variance increases, according to a Neyman allocation; however monthly efforts consistently range from 8 to 13 net sets. Both large and small mesh gillnets were set near each other such that they never intersect or connect, but can be observed simultaneously by sampling crew. Locations of sample sites are shown in Supplementary Fig. S1. A two-tailed t-Test assuming unequal variance indicated that there were no significant differences between annual catch per unit effort (CPUE), calculated as captured individuals/gillnet hours, of bull sharks caught in small, perpendicular-set nets vs. large, parallel-set nets (p = 0.31, t = 1.03). Thus, CPUE from both nets was combined for analysis. Sharks were measured to fork length. Environmental conditions including date, time, soak time, tide (high or low), depth, set type, and GPS coordinates were recorded during the soak. Water quality data including salinity (ppt), dissolved oxygen (mg/L), and temperature (°C) were also collected on the surface at the midpoint of the gillnet using a YSI 85.

### Analysis

Coastal Change Analysis Program (C-CAP) data was used to obtain LU/LC area coverage for the Mobile Bay watershed, with values available for the years 2001, 2006, 2011, and 2016^38^. These LU/LC values were linked to catch records based on proximate sampling years as temporal resolution of LU/LC allowed (e.g., a shark captured in 2003 would be linked to 2001 LU/LC data, while a shark captured in 2004, would be linked to 2006 LU/LC data). Riverine discharge values were retrieved from USGS gages located at Claiborne and Coffeeville, AL, which mark the upper bounds of the estuary^36^. Riverine discharge values were linked to catch data records based on mean monthly values. Monthly Chl-a values were extracted as raster data (4 km spatial resolution) from the MODIS-Aqua satellite, and linked to catch data by spatial overlap and monthly values.

Time series analysis of annual CPUE were conducted using the ‘lm’ function, which conducts linear regressions, in the base package ‘Stats’ in R^39^. Trends were considered significant at p < 0.05. Individual univariate regressions were conducted for the effects of year on mean annual values of in situ (i.e., depth, SST, dissolved oxygen, and salinity) and retrieved (i.e., LU/LC, riverine discharge, and Chl-a) data to identify potential changes in environmental conditions throughout the study period (Table 2). Residuals of the data were assessed for normality using a Shapiro-Wilks test.

Boosted Regression Tree models were fitted for bull shark occurrence (i.e., presence-absence) to develop Species Distribution Models (SDMs) using a total of seventeen variables (Table 1). Boosted regression trees are an additive regression tree model in which models are composed of simple regression trees and built upon an increasing number of iterative trees until the collective model error is minimized and an optimal tree is created. These models are powerful because they are robust to collinearity, missing data, outliers, and can accommodate nonlinear trends^40^. The response variable was binary: presence (1) or absence (0) of a bull shark, therefore the BRTs were built using a Bernoulli distribution and can be interpreted as a probability of presence. Because these SDMs are built on the assumption that environmental conditions at the time and location of a capture indicate suitable habitat, they can be interpreted as habitat suitability models. Boosted regression trees were developed iteratively with the ‘gbm.step’ function in the ‘dismo’ package^41^ based on different combinations of learning rate (LR), bag fraction (BF), and tree complexity (TC) model parameters^42^. Learning rate describes the proportion of influence for each individual tree term, bag fraction is the proportion of testing data used to train the model, and tree complexity is the number of nodes on an individual tree. Models were fitted in iterations of BF = (0.5, 0.7, 0.9), LR = (0.01, 0.005, 0.001, 0. 000001), and TC = (2, 7, 8). The final model was selected to maximize Cross-Validation Area Under the Receiver Operating Curve (CV-AUC), minimize standard error, and maximize Training Data AUC (TD-AUC)^44^. R code was guided by Elith & Leathwick^43^. After selection of final model parameters, the model was run again to include a random predictor variable, which was populated using the “RAND” function in Excel. The explanatory power of the random variable served as a threshold for predictive performance, and variables yielding values below the predictive power of the random variable were not reported and considered insignificant predictors.

**Table 1:** Data source, mean values and ranges for potential environmental predictors used in training boosted regression trees.

Predictor	Source	Unit	Mean ± SD	Range
Temperature	AMRD	°C	25.78 ± 5.35	0.30–34.70
Depth	AMRD	ft	3.50 ± 1.47	0–55.00
Salinity	AMRD	ppt	13.56 ± 8.14	0–34.00
DO	AMRD	mg/L	6.93 ± 1.85	0.90–18.40
Riverine discharge	USGS	m^3^/s	1609.68 ± 1330.47	145.18–6160.33
High intensity developed	C-CAP (NOAA)	sq. miles	22.23 ± 1.70	20.77–24.15
Low intensity developed	C-CAP (NOAA)	sq. miles	62.02 ± 2.51	59.76–64.52
Open space developed	C-CAP (NOAA)	sq. miles	28.04 ± 1.81	26.45–29.92
Grassland herbaceous	C-CAP (NOAA)	sq. miles	13.98 ± 0.37	13.46–14.33
Agriculture	C-CAP (NOAA)	sq. miles	127.95 ± 1.54	126.15–129.33
Forested	C-CAP (NOAA)	sq. miles	90.16 ± 1.10	88.79–91.13
Scrub	C-CAP (NOAA)	sq. miles	19.98 ± 2.01	18.21–21.89
Woody wetland	C-CAP (NOAA)	sq. miles	91.39 ± 1.45	90.12–92.85
Emergent wetland	C-CAP (NOAA)	sq. miles	16.37 ± 0.47	15.87–16.98
Bare land	C-CAP (NOAA)	sq. miles	8.36 ± 0.25	8.03–8.57
Open water	C-CAP (NOAA)	sq. miles	392.52 ± 0.12	392.41–392.67
Chl-a	MODIS-Aqua (NASA)	mg/m^3^	58.82 ± 42.48	0.00–157.58

AMRD is the Alabama Department of Conservation and Natural Resources-Marine Resource Division, which collected data in situ.

Data collected in situ in 2003–2005 and 2018–2020 were rasterized via ordinary Kriging at a resolution of 500 m, which is the approximate width of the narrowest sampling area (Supplementary Figs. S2–S5). This resolution allowed the shape of the sampling areas to be maintained when rasterized for distribution modeling. Given the extensive spatiotemporal sampling coverage along the coastline (Supplementary Figs. S2–S5), points sampled at different seasons should have similar relative influence on a cell value. These raster layers were joined with mean annual LU/LC values, riverine discharge, and Chl-a from their respective years to comprise two temporally distinct predictor groups. The years 2003–2005 were selected to represent the early portion of the time series because 2003 was the earliest year that Chl-a data was available, while the years 2018–2020 were the most recently completed years of sampling at the start of analysis. Predictive data were grouped to reduce interannual variability. Rasterized mean annual data for the years 2003–2005 and 2018–2020 were used as input variables in the optimal boosted regression tree model using the ‘predict’ function in the aforementioned ‘dismo’ package. This yielded two respective habitat suitability (or probability of occurrence) maps that reflect early and recent portions of the time series. These methods were guided by best practices according to Hijmans and Elith^42^ and all analyses were conducted using R version 4.3.1^39^. Using the ‘gbm.loop’ function in the ‘gbm.auto’ package^40^, coefficients of variation, representative of model error, were calculated for each raster cell in the habitat suitability maps.

## Results

**Figure 2:**
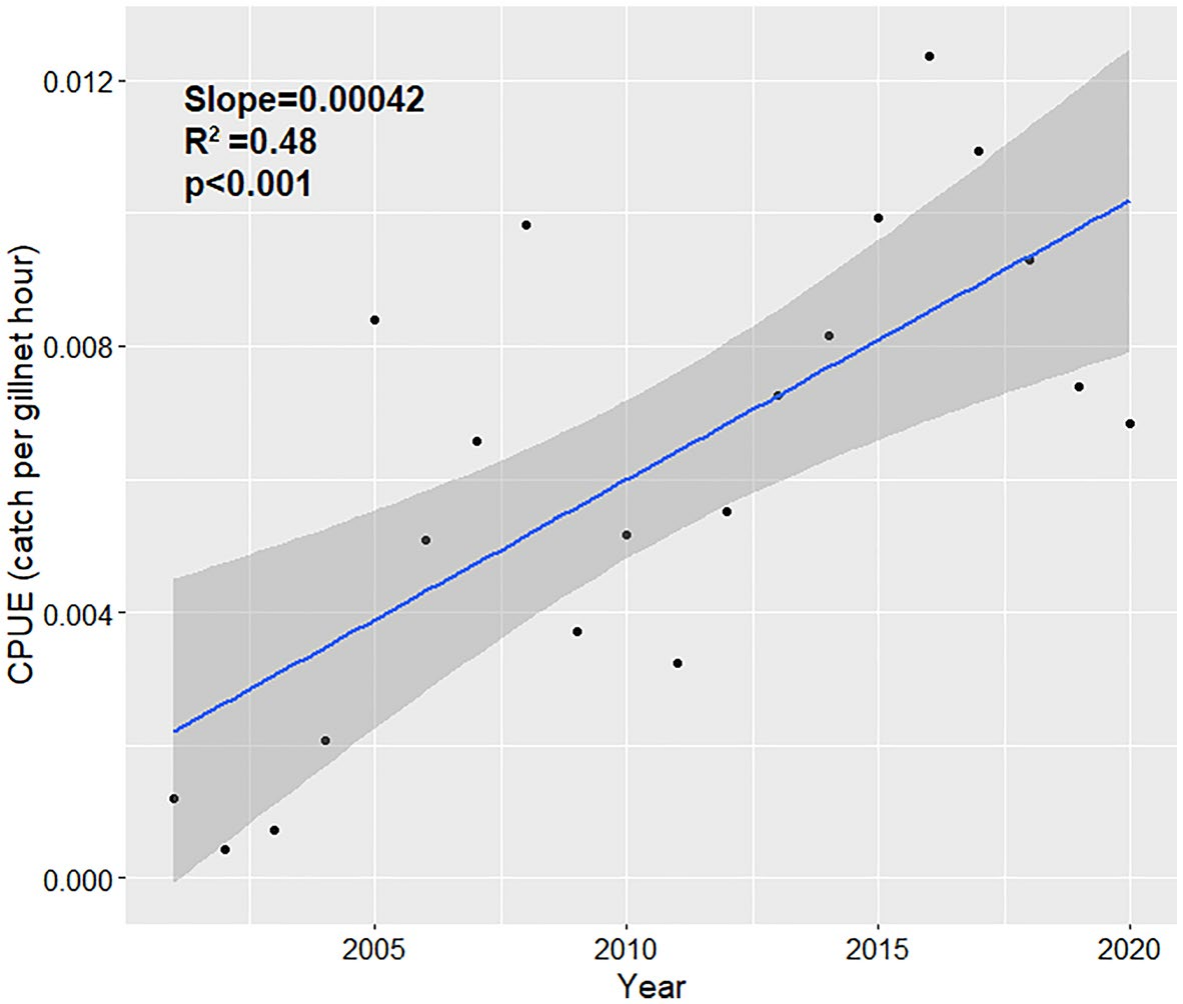
Catch-per-unit-effort of juvenile bull sharks per year. The dark grey region indicates standard error.

**Figure 3:**
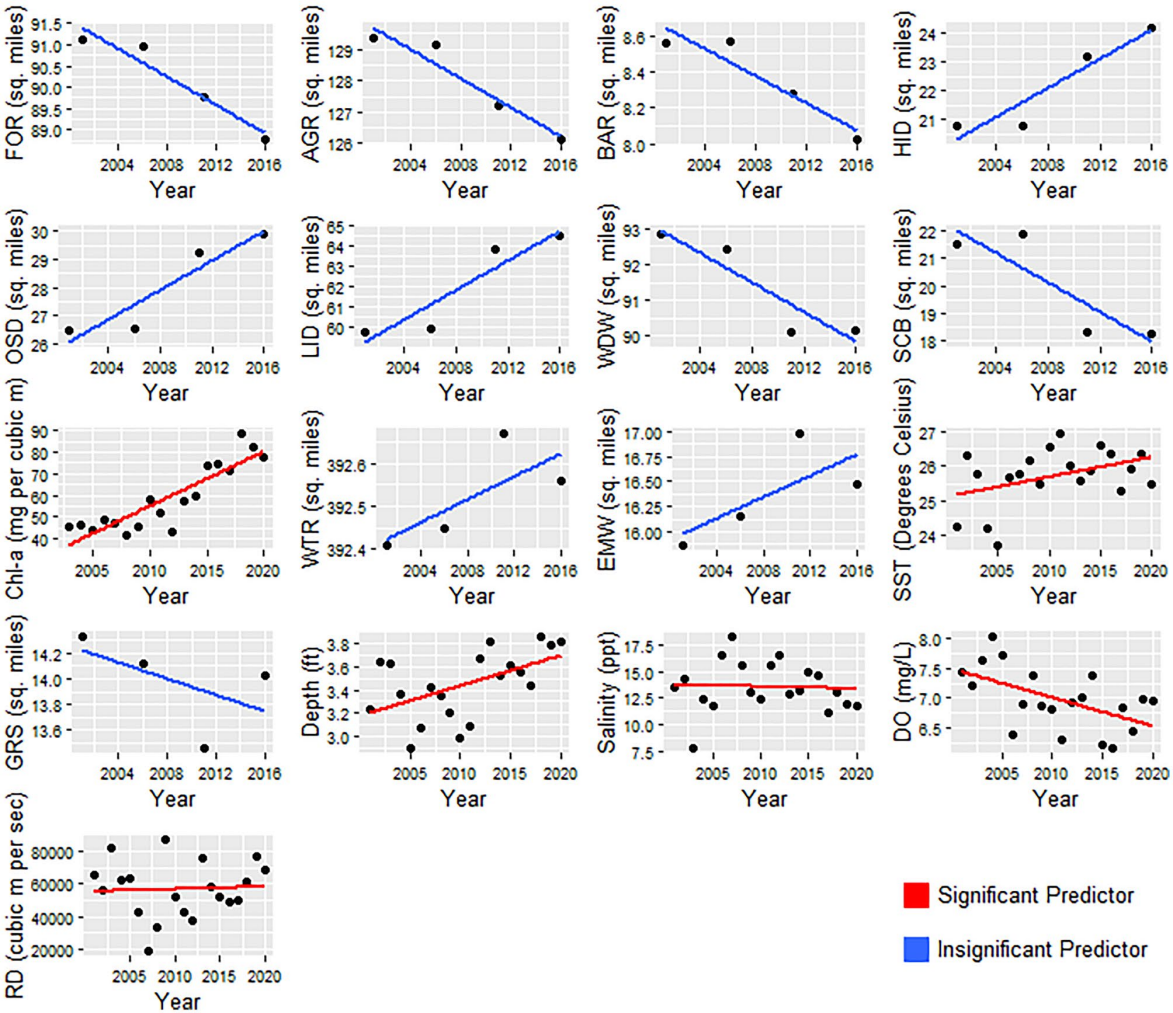
Scatterplot of annual environmental parameters with fitted linear regressions. Variables considered to be significant predictors according to BRT output are designated with red regression lines and those considered insignificant with blue regression lines. LU/LC data only have four available values during the study timeframe and represent exact measurements. All other data are mean annual values. The C-CAP categories for LU/LC include forested (FOR), agriculture (AGR), bare land (BAR), high intensity developed (HID), open space developed (OSD), low intensity developed (LID), woody wetland (WDW), scrub (SCB), open water (WTR), emergent wetland (EMW), and grassland herbaceous (GRS). RD represents riverine discharge.

The gillnet dataset included 440 bull sharks caught from 2003 to 2020. Bull shark fork length ranged from 335 to 1068 mm, indicating that all captured individuals were immature^44^. Catch per unit effort significantly increased over five-fold during the 20-year study period from 0.0012 individuals/gillnet hrs to 0.0068 individuals/gillnet hrs (Fig. 2). Concomitantly, mean SST rose from 22.3  °C in 2001 to 23.0  °C in 2020. Overall annual SST increased at a mean rate of 0.05 °C/yr (Table 2), which aligns with the rates observed by Turner et al.^29^. There were also substantial decreases over time in forested (FOR), agriculture (AGR), bare land (BAR), woody wetland (WDW), and Scrub/Shrub (SCB) land cover, and increases in high-intensity development (HID), open-space development (OSD), and low-intensity development (LID) land cover, as well as Chl-a and SST (Table 2, Fig. 3).

**Table 2:** Time series regression results of environmental parameters.

Variable	Unit	Slope (Unit/Year)	R^2^	P	Std. error
Forested*	sq. miles	− 0.15	0.89	< 0.001	0.33
Agriculture*	sq. miles	− 0.21	0.87	< 0.001	0.49
Bare*	sq. miles	− 0.04	0.86	< 0.001	0.09
High intensity developed*	sq. miles	0.23	0.85	< 0.001	0.60
Open space developed*	sq. miles	0.20	–	0.004	0.06
Low intensity developed*	sq. miles	0.27	–	0.006	0.09
Woody wetland*	sq. miles	− 0.14	–	0.03	0.06
Scrub	sq. miles	− 0.19	–	0.05	0.09
Chl-a	mg/m^3^	2.32	–	0.002	0.62
Open water*	sq. miles	0.01	0.38	< 0.01	0.08
Emergent wetland*	sq. miles	0.03	–	0.04	0.48
Sea surface temperature	°C	0.06	0.12	0.07	0.78
Grassland herbaceous*	sq. miles	− 0.02	–	0.04	0.69
Depth	ft	− 0.04	0.04	0.19	0.88
Salinity	ppt	0.2	0.03	0.23	5.50
Dissolved oxygen	mg/L	− 0.03	− 0.03	0.49	1.14
Riverine discharge	m^3^/s	150.9	–	0.87	914.4

Data reflect mean annual changes from the year 2000 to 2020, with the exception of Chl-a sourced from the MODIS satellite, which begins in 2003, and C-CAP data (indicated by *), which begins in 2001 and ends in 2016 and is recorded every 5 years.

Variables with missing R^2^ values were calculated as generalized least squares models to account for temporal autocorrelation. All other variable metrics are reported from ordinary least squares models.

**Figure 4:**
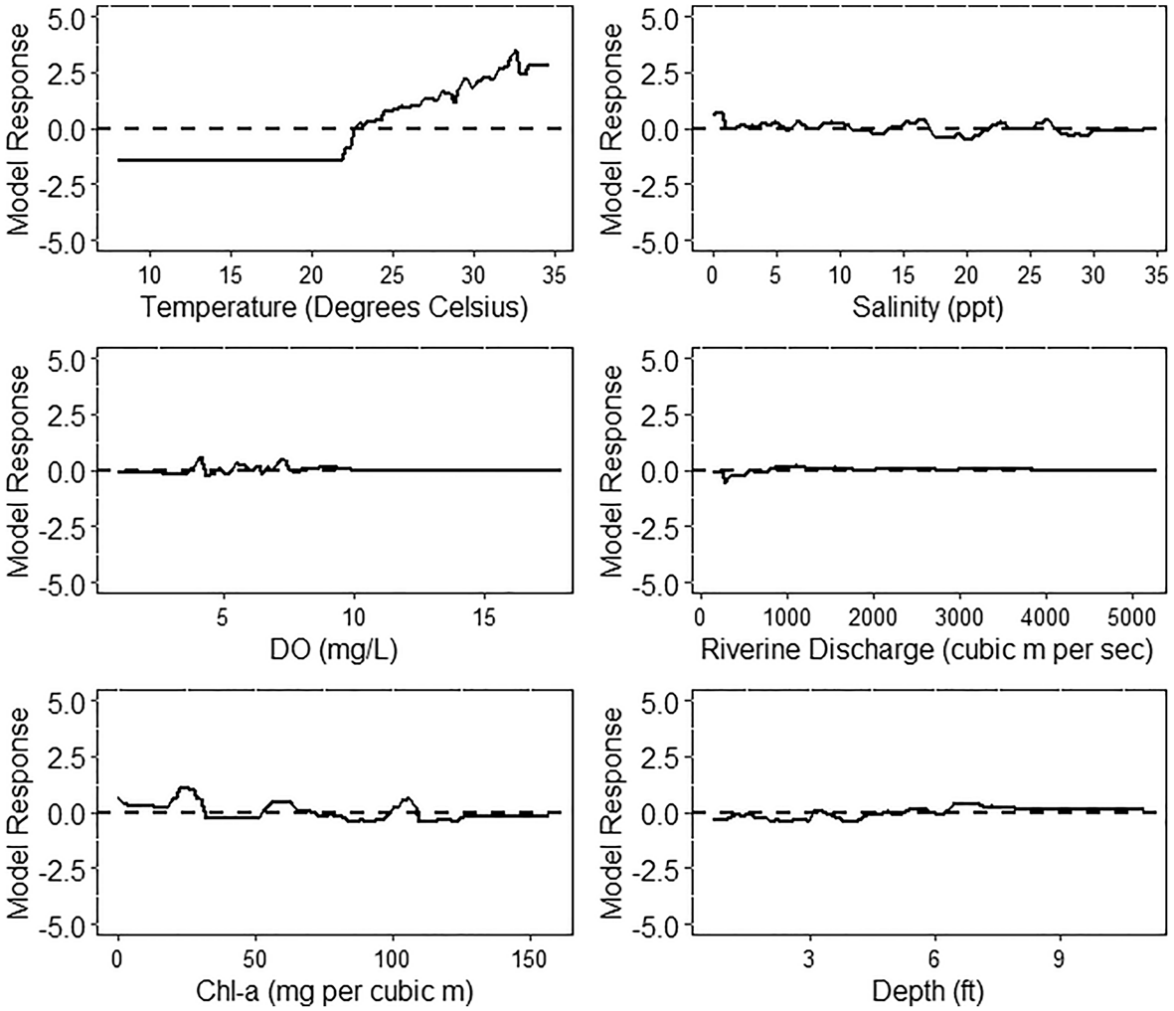
Line plots displaying the marginal effects of predictors performing better than random for bull sharks derived from boosted regression trees. The y-axis is a logit scale, where values of 0 indicate a random likelihood of bull shark presence, values > 0 indicate increased probability, and values < 0 indicate reduced probability.

The final BRT model for bull sharks yielded a CV-AUC of 0.858, suggesting “very good” model fit according to criteria established in Lane et al.^45^. Sea surface temperature accounted for the most variability in suitable bull shark habitat at 27.6%, followed by salinity at 18%. Riverine discharge, Chl-a, and depth were comparable, at 11.6%, 10.6% and 9.4% respectively. SST conveyed the most apparent relationship with model response, with SSTs above *ca*. 22.5 °C resulting in a positive likelihood of bull shark presence (Fig. 4). This likelihood increased in magnitude with warmer SST and exhibited no maximum thermal threshold (Fig. 4). Observable trends are less clear for the other variables. When incorporated into the final model, the random variable yielded an explanatory power of 8.9%. As such, the marginal effects of riverine discharge, Chl-a, and depth indicate they contributed to model performance only somewhat more than random.

**Figure 5:**
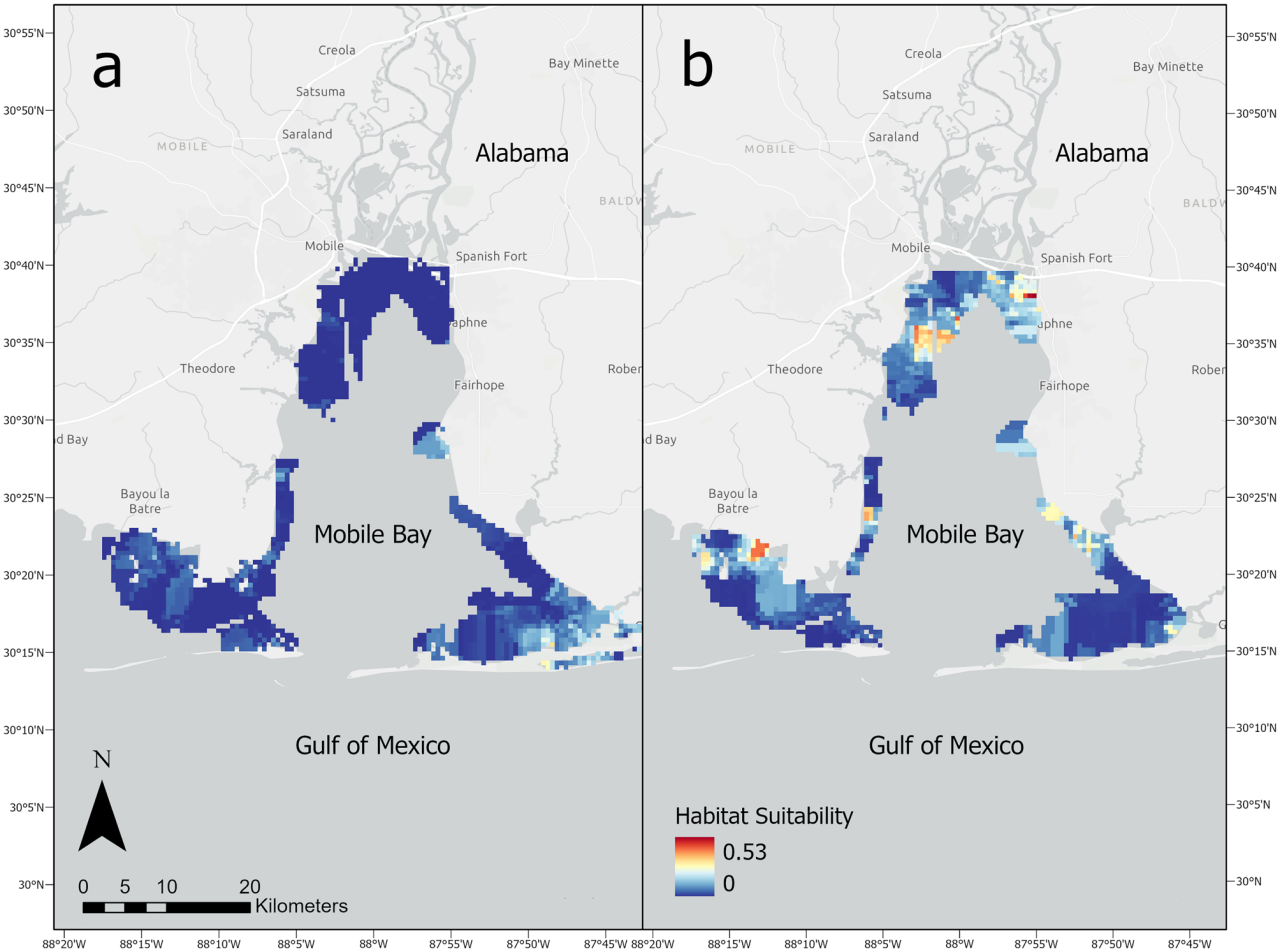
Species distribution model for bull shark derived from boosted regression trees, which predicts suitability of habitat based on environmental data sourced from 2003 (**a**) and 2020 (**b**).

**Figure 6:**
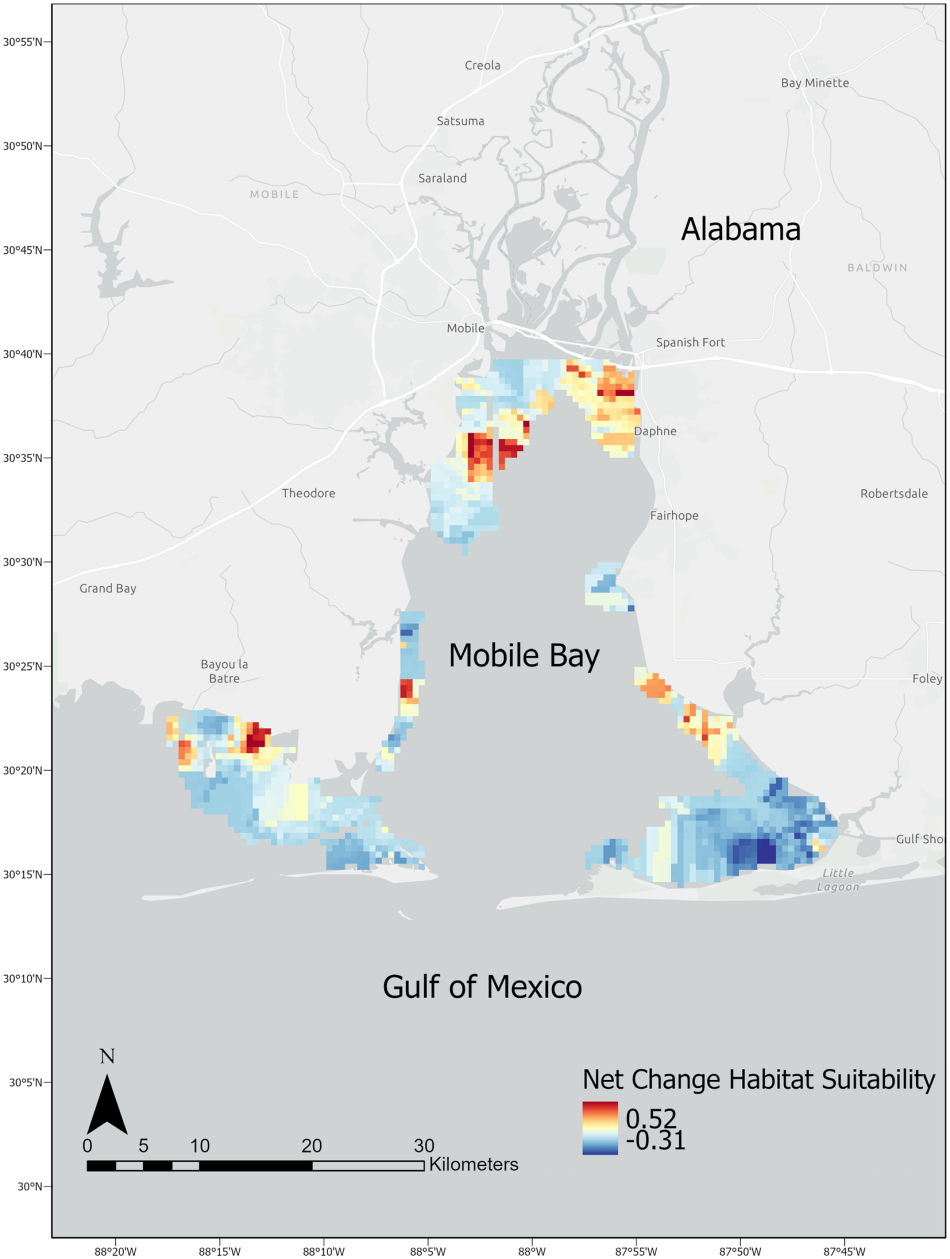
Net change in predicted habitat suitability from 2003 to 2020 for bull sharks.

Species distribution model results from 2003 indicated greatest habitat suitability in the southwest and southeast portions of the study area as far north as Daphne, AL (Fig. 5a). However, habitat suitability exhibited limited variability across the study area in 2003. By contrast, SDM results from 2020 indicated a wider range of habitat suitability, with least suitable habitat located in the southeast portion of the study area, and most suitable habitat concentrated near Daphne and along the western shoreline of the bay (Fig. 5b). Changes in net predicted relative abundance from 2003–2020 indicated increases in mean habitat suitability that align with the early locations of greatest suitability that expanded in area and magnitude in 2020 (Fig. 6). Mean habitat suitability increased from 0.028 ± 0.039 in 2003–2005 to 0.081 ± 0.087 in 2018–2020. The observed trend in CPUE aligns with the results of the species distribution models yielded by the BRT analysis, which indicate both an increase in the magnitude and geographic extent of habitat suitability for juvenile bull sharks on the Alabama coast over the last 20 years (Fig. 5). Coefficient of variation for model predictions were low (< 1%), and did not appear to exhibit any spatiotemporal patterns (Supplementary Fig. S6a, b).

## Discussion

Using long-term gillnet sampling, remote sensing and spatiotemporal modeling, this study documents changes in habitat suitability of juvenile bull sharks in a suspected Gulf of Mexico nursery habitat amidst the changing seascape of the Anthropocene^2^. Results indicate that increasingly warm SST off the coast of Alabama has coincided with increases in bull shark abundance and habitat suitability, with consequences for future habitat suitability in this region. Given the magnitude of the effects of SST on the SDM, as well as the apparent trend of SST over the study period, we deduced that of the variables tested, increases in SST are primarily responsible for the observed change in juvenile bull shark habitat suitability. This study illuminates the role of increasing temperatures in expanding suitable habitat for bull sharks in the northern Gulf of Mexico.

Adaptation occurs at evolutionary time scales, which many forecasters predict will not keep pace with rising temperatures associated with climate change for long-lived species like sharks^46^. However, shifts in animal behavior and distributions can be much more responsive, and may enable mobile species to adjust to changing conditions. Range expansions of the juvenile bull shark have been documented in the northwestern Atlantic as a response to increasing SST, demonstrating the ability of this species to alter their habitat use as such^14^. This study showcases how the use of an estuarine habitat in the northern Gulf of Mexico, a location central to their latitudinal range, has not only persisted, but perhaps been enhanced, in conjunction with warming waters. The lack of an upper SST threshold up to 34.7 °C suggests thermal tolerance for juvenile bull sharks in this area exceeds the thermal maximum of 30 °C postulated by Drymon et al.^23^ for the region, but does fall within the range of preferred SST of 27–37.3 °C identified for juveniles near southern Florida^47^. These findings also align with those of Curtis et al., who identified no thermal maximum and a preference for water temperatures > 20 °C for juveniles on the Atlantic coast of Florida^48^. Other studies in the Gulf of Mexico found either no significant effect or a positive correlation with temperatures of 24–26 °C for adult bull shark distributions, both of which contrast with the observed trends for juvenile bull sharks^49,50^. The difference in preferred temperature ranges between juveniles and adults may indicate different physiological constraints in the face of thermal pressures, suggesting the effects of climate change on species may vary based on life history stage. Further, changes in habitat use may also vary ontogenetically, as juveniles may be forced to occupy less suitable habitat to avoid larger conspecifics. Thermal metabolic tolerance of juvenile bull sharks has been found to exceed 30 °C^51^, suggesting juvenile thermal tolerance can be robust to warmer temperatures, allowing them to occupy unique habitat. As such, future research into the effects of increasing temperatures on different life and reproductive phases is merited.

Contrary to the grim global outlook forecasted for many shark species^2,8^, this study illuminates the resiliency and potential benefits rendered for juvenile bull sharks in the northern Gulf of Mexico in the face of climate change and coastal urbanization. As of the publication date, the bull shark stock in the Gulf of Mexico is unassessed and undergoing its first stock assessment through the Southeast Data, Assessment and Review (SEDAR) process. Our findings are indicative of a local bull shark population that is persisting and perhaps growing, similar to that reported in the western Gulf of Mexico by Froeschke et al.^52^. However, while species distribution studies rely heavily on the assumption that conditions at the time and location of capture reflect suitable habitat for a species, they generally do not consider the condition of the animal. Warming waters have been shown to impose stresses such as increased feeding demands and aerobic metabolism on sharks^53^; therefore, juvenile bull sharks in Mobile Bay will likely face increasing metabolic demands as waters continue to warm. Increased feeding propensity can be met with increased risk^17^. However, it currently appears that the benefits of increased temperatures, such as decreased development time and expanded habitat suitability, outweigh the costs. In fact, the region in the current study experiencing the greatest increases in habitat suitability also has low hydrological residence times and optimal salinity ranges^54^, conditions which contribute to a highly productive ecosystem^55^. Should a temperature threshold be exceeded though, understanding how populations may respond is of critical importance for management. At present, migrations of bull sharks tagged off Alabama have not surpassed the Florida Keys^50^, and a northward expansion to alleviate thermal pressures would require an extensive expansion of this population’s migratory range. To avoid the energetic demands and predation risks associated with extensive migrations, it is possible that bull sharks may spend increased time in riverine habitat in search of cooler waters, conditions which would not necessarily be physiologically limiting for the bull shark over an extended period of time^56^. Continued research into the physiological limits and migratory capacity of this population are warranted.

The recovery and management of elasmobranchs requires a multifaceted approach, which includes enacting spatial protections of critical habitat^57^. Species distribution modeling is a tool that can be used to delineate critical habitat, and has been used to assess the effectiveness of spatial protections for highly migratory species such as bull sharks^18^. Because their predictive power relies upon a set of environmental criteria unique to a species, they can also be used to anticipate changes in distribution in response to hypothetical changes in environmental conditions, such as those induced by climate change. Under the assumption that species relationships to environmental conditions are held constant, they can be used to develop proactive rather than reactive management strategies that have historically characterized shark management^58^. Climate change is listed as a significant threat for sharks, and when coupled with pressures from overfishing, could drive many shark species to more extreme categories of the IUCN Red List by 2050^8,59^. In the Gulf of Mexico, the overfishing^60^ and invasive species^61^ currently threatening coastal ecosystems may be sufficiently exasperated by climate change to impose additional stresses on this subpopulation’s habitat suitability prior to the breach of a physiological thermal maximum. As an example of successful sustainable shark fisheries management^11,62^, the United States will need to employ a dynamic and flexible strategy to continue the legacy of success in shark management^6^. Accordingly, these kinds of spatial analyses may be a useful tool to inform fisheries monitoring and management in their task of keeping pace with the pressures of climate change.

Despite the development that has occurred on the Alabama coast over the past several decades^31^, land-use/land-cover changes were not identified as significant predictors of habitat suitability. Given the increase in juvenile bull shark abundance in the area, it appears that they are not deterred from occupying habitat near urbanized coastline in the northern Gulf of Mexico. This aligns with the findings of Hammerschlag et al.^63^, in which sharks did not actively avoid urban areas near Miami, FL. With increasing abundances of bull sharks near the Alabama coastline, increases in human-wildlife interactions are a likely consequence. Unlike the ‘Jaws Effect’ that has historically plagued shark management initiatives, surveys of recreational fishermen find that the impacts they perceive sharks will have on fishing opportunities are what primarily hinder their willingness to support shark protection and conservation initiatives^64^. One example of such an impact is through depredation, defined as the “removal of a hooked fish from an angler’s line”^64^. Given that bull sharks have been identified as common depredators in the north-central Gulf of Mexico^65^, these changes could hinder stakeholder willingness to support shark conservation and management plans. With a dwindling commercial and increasing recreational shark fishery in the United States, recreational fishermen have the capacity to influence the success and monitoring of shark management plans in the United States through means such as monitoring, engagement in research, enforcement, and advocacy^66^. As such, addressing the concerns of these fishermen and educating them on the significant ecological role sharks play in the health of estuarine systems should be a priority.

To develop a species distribution model (SDM) that included parameters beyond those collected in situ, this study outsourced additional environmental parameters (i.e., Chl-a, riverine discharge, and LU/LC) to develop a model intended to characterize the changes undergone by the Alabama coast over the past two decades. These parameters were available at coarser spatiotemporal resolutions than the in-situ data and were used as monthly and annual mean values. This weakens the signals of these variables when training the models, especially in contrast with the in-situ data. For example, daily and monthly Chl-a was identified as a significant predictor of bull shark distributions by Rider et al.^50^, but was insignificant when Calich et al. used seasonal and annual values like our study^67^. Although it was considered a top predictor for our model, the lack of a clear relationship suggests that the temporal resolution may not be appropriate for this scope of analysis. Since Chl-a is used as a proxy for primary productivity and by relation, prey availability, the sensitivity of bull shark response to this variable likely depends on how closely the temporal resolution aligns with the movements of their prey. On the other hand, bias introduced by using rasters developed from the in-situ variables as predictors can influence the SDM to reflect conditions at the time of sampling rather than overall mean conditions. While sampling occurs year-round, increased frequency of sampling in warmer months may skew the input rasters and thus the habitat suitability models to more closely reflect the well-mixed, low salinity conditions of Mobile Bay in the summer^36^. Future studies must consider the balance between the benefits of incorporating data sourced from in situ recordings, which offer a more precise understanding of the relationship between species occurrence and environmental parameters when training models, with remotely sensed data, which may reduce bias and provide a more accurate view of overall mean annual conditions when projecting species distributions.

Bull sharks depend on nursery habitats for maturation, as they provide ample prey and reduced mortality during an energetically costly and vulnerable life stage^24^. As such, changes in habitat use of juvenile bull sharks in a suspected nursery area like Mobile Bay are likely to have rippling effects on the persistence of a population^24^. The findings of this study suggest that at SST above 22.5 °C, juvenile bull sharks are resilient and may yield a net benefit from large-scale changes wrought by climate change. However, unlike populations in the Atlantic^14^, the Alabama population of bull sharks faces geographic constraints that may cause them to be uniquely vulnerable should a thermal threshold be exceeded. Further, changes in apex predator abundance may have significant ecological and social consequences, altering the structure of the community through predation and risk mediation behavior of prey, while leading to increased human-wildlife interactions^64,68^. Continued monitoring of the local population is imperative for assessing the scope of these potential impacts and when coupled with species distribution modeling, can be used to anticipate changes in distribution due to the impacts of climate change, equipping managers with flexible and proactive means necessary to mediate the impacts of climate change.

### Supplementary Information

Supplementary Legends.

Supplementary Figure S1.

Supplementary Figure S2.

Supplementary Figure S3.

Supplementary Figure S4.

Supplementary Figure S5.

Supplementary Figure S6.

## Data Availability

The datasets analyzed during the study are available in Dryad at https://datadryad.org/stash/share/HrSKF7glzqiGbqfk5u9zwXA2pDNlhgIeG2wpe1iT0gQ.
