# Supplementary material for: Warming waters lead to increased habitat suitability for juvenile bull sharks (Carcharhinus leucas)
Source: Sci Rep. 2024 Mar 14;14:4100. doi: 10.1038/s41598-024-54573-0 (PMC10940676; doi:10.1038/s41598-024-54573-0)
Supplement: Supplementary file 1 — Supplementary Legends. [file 41598_2024_54573_MOESM1_ESM.docx]

Supplementary Figure S1. Locations of gillnet set midpoints conducted by the Alabama Department of Natural Resources from 2003-2020.

Supplementary Figure S2. Sea surface temperature (SST) raster layers yielded by kriging, which were then used as predictor surfaces for habitat suitability modeling representative of the years 2003-2005 (S2a) and 2018-2020 (S2b). Locations of in situ measurements for each respective year group are superimposed on the raster layers.

Supplementary Figure S3. Salinity raster layers yielded by kriging, which were then used as predictor surfaces for habitat suitability modeling representative of the years 2003-2005 (S3a) and 2018-2020 (S3b). Locations of in situ measurements for each respective year group are superimposed on the raster layers.

Supplementary Figure S4. Dissolved oxygen raster layers yielded by kriging, which were then used as predictor surfaces for habitat suitability modeling representative of the years 2003-2005 (S4a) and 2018-2020 (S4b). Locations of in situ measurements for each respective year group are superimposed on the raster layers.

Supplementary Figure S5. Depth raster layers yielded by kriging, which were then used as predictor surfaces for habitat suitability modeling representative of the years 2003-2005 (S5a) and 2018-2020 (S5b). Locations of in situ measurements for each respective year group are superimposed on the raster layers.

Supplementary Figure S6. Coefficient of variation of presence-absence (e.g., habitat suitability) values yielded from BRT models for year groups (a) 2003-2005 and (b) 2018-2020.
