# Supplementary figures and images for: Warming waters lead to increased habitat suitability for juvenile bull sharks (Carcharhinus leucas)
Source: Sci Rep. 2024 Mar 14;14:4100. doi: 10.1038/s41598-024-54573-0 (PMC10940676; doi:10.1038/s41598-024-54573-0)

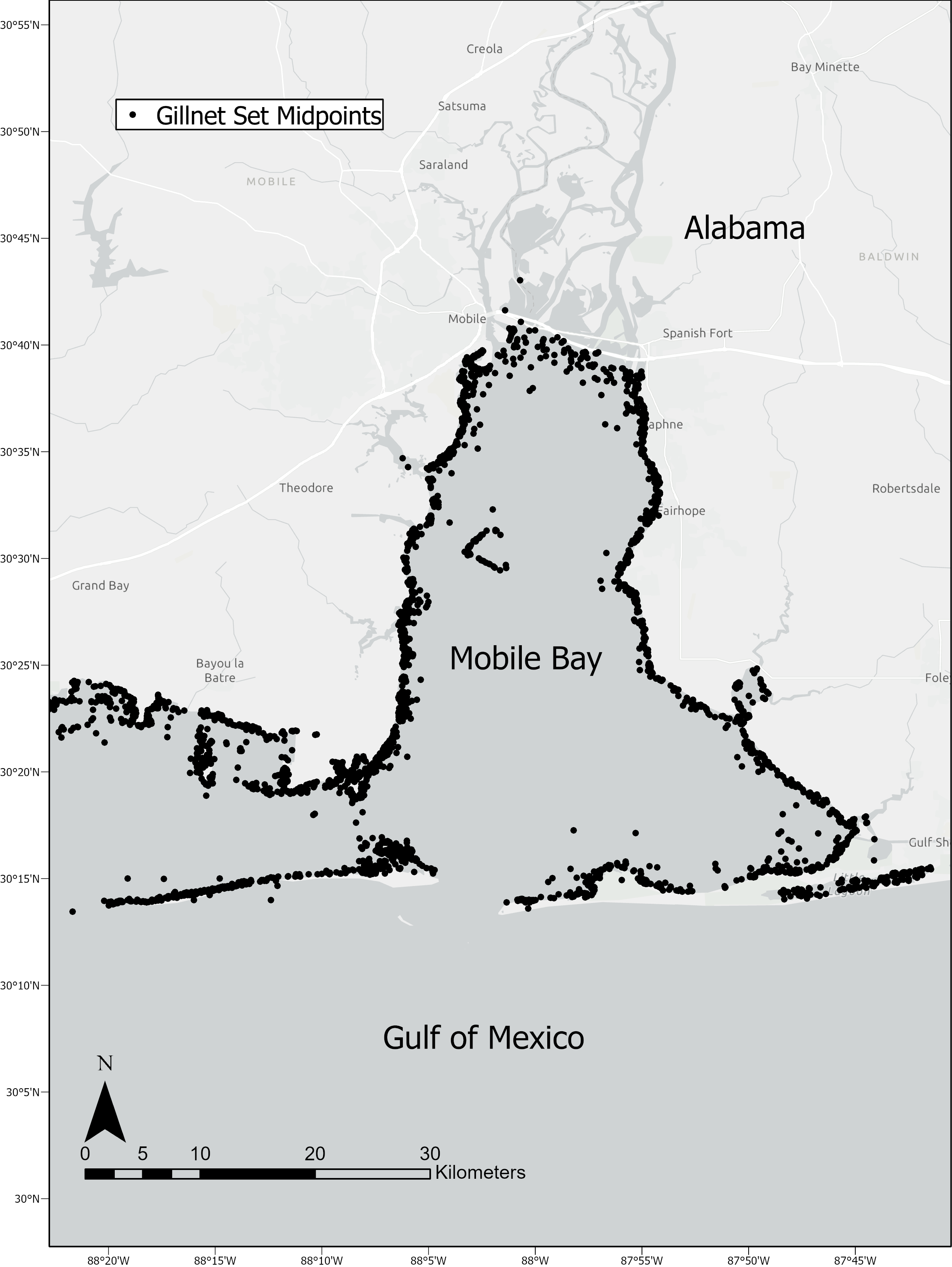

Supplement: Supplementary file 2 — Supplementary Figure S1. [file 41598_2024_54573_MOESM2_ESM.png]

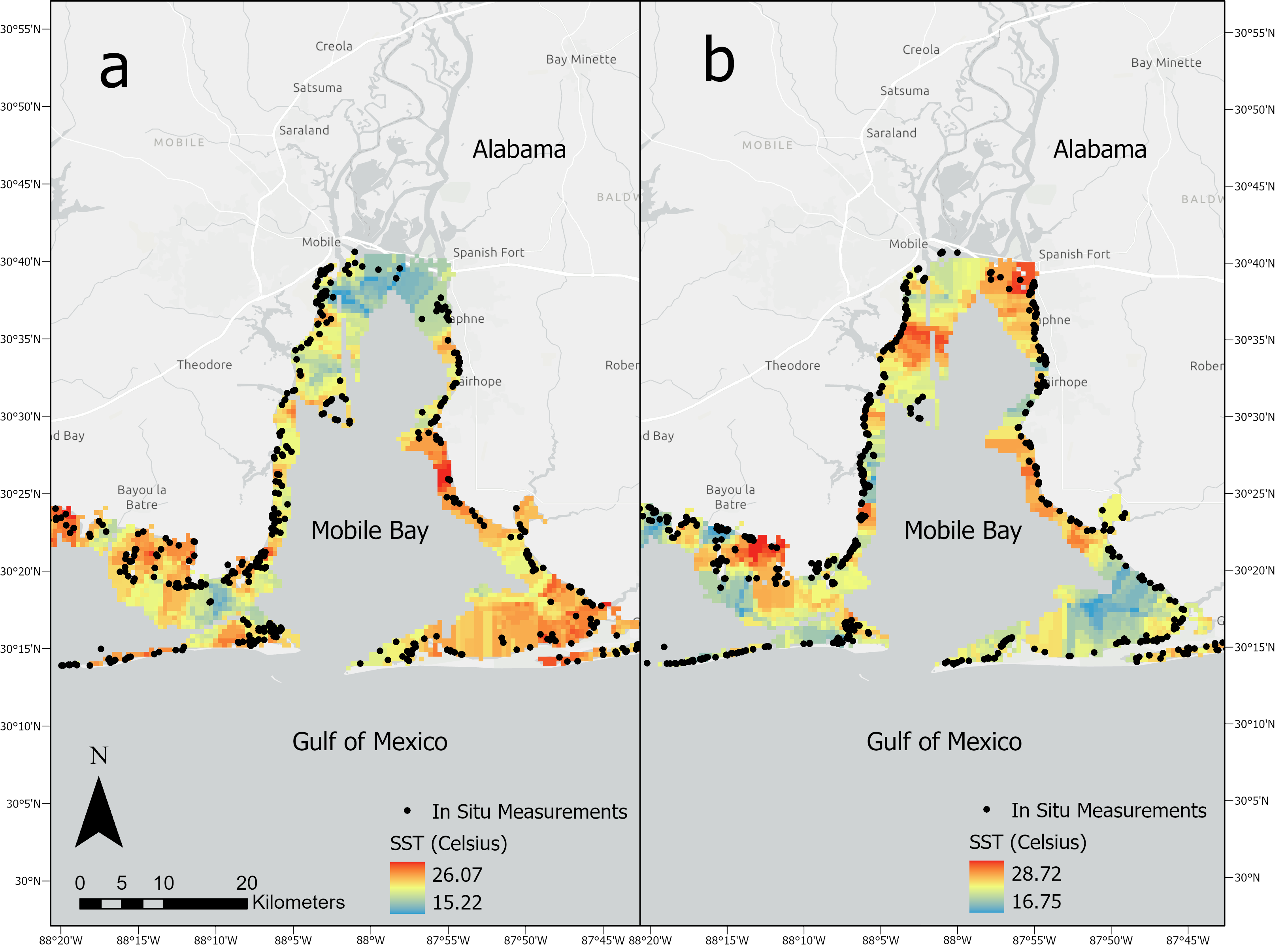

Supplement: Supplementary file 3 — Supplementary Figure S2. [file 41598_2024_54573_MOESM3_ESM.png]

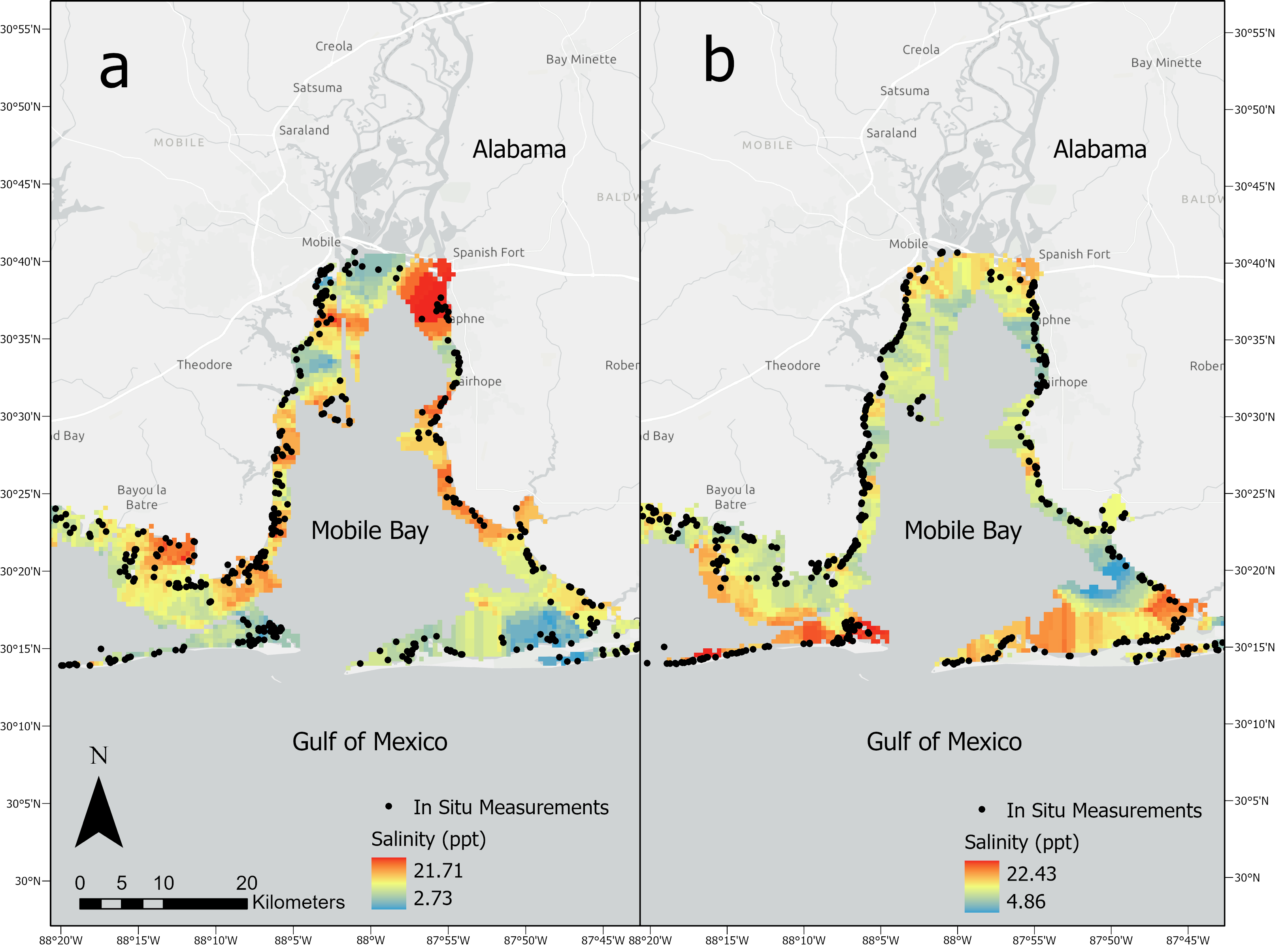

Supplement: Supplementary file 4 — Supplementary Figure S3. [file 41598_2024_54573_MOESM4_ESM.png]

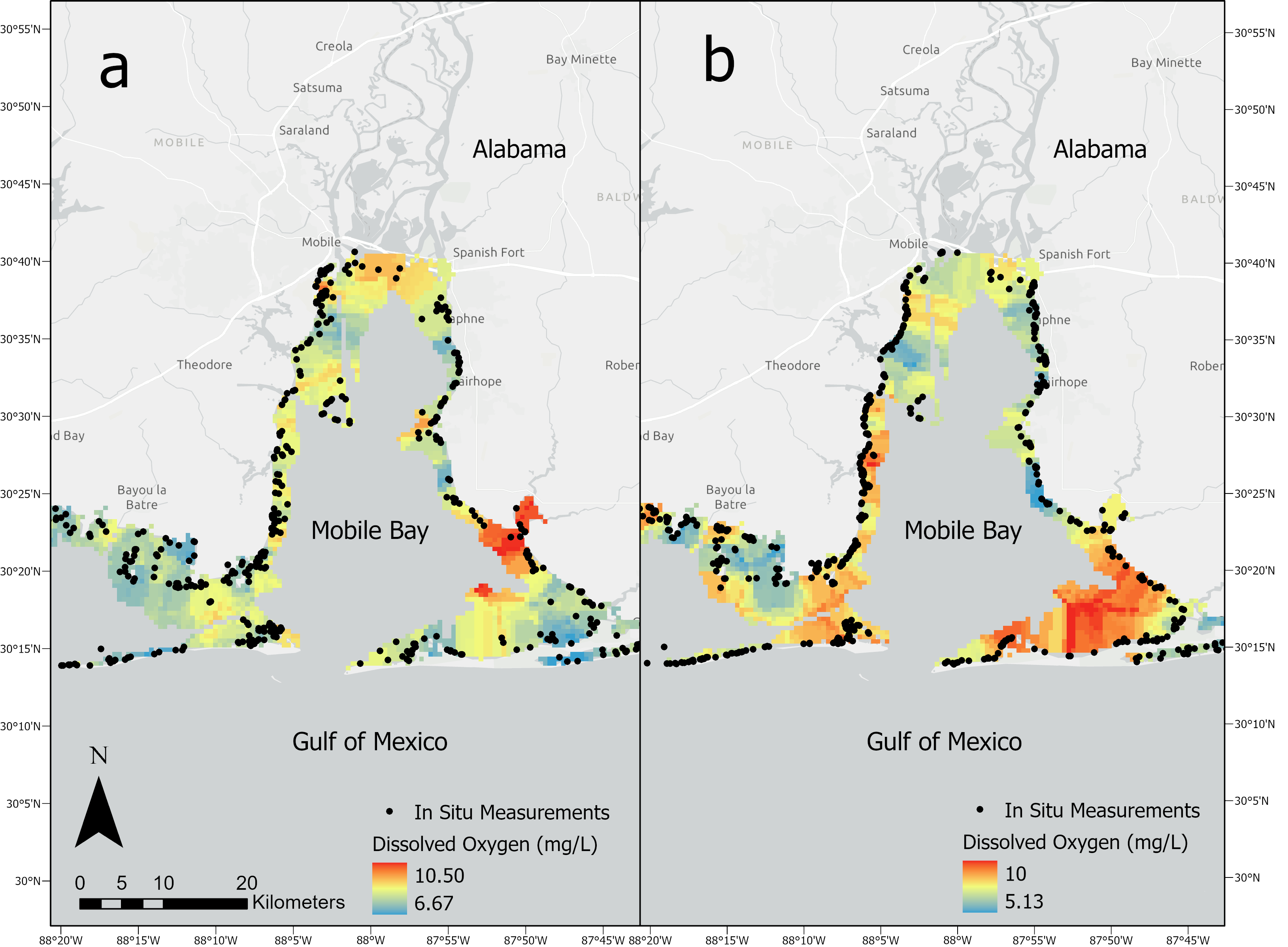

Supplement: Supplementary file 5 — Supplementary Figure S4. [file 41598_2024_54573_MOESM5_ESM.png]

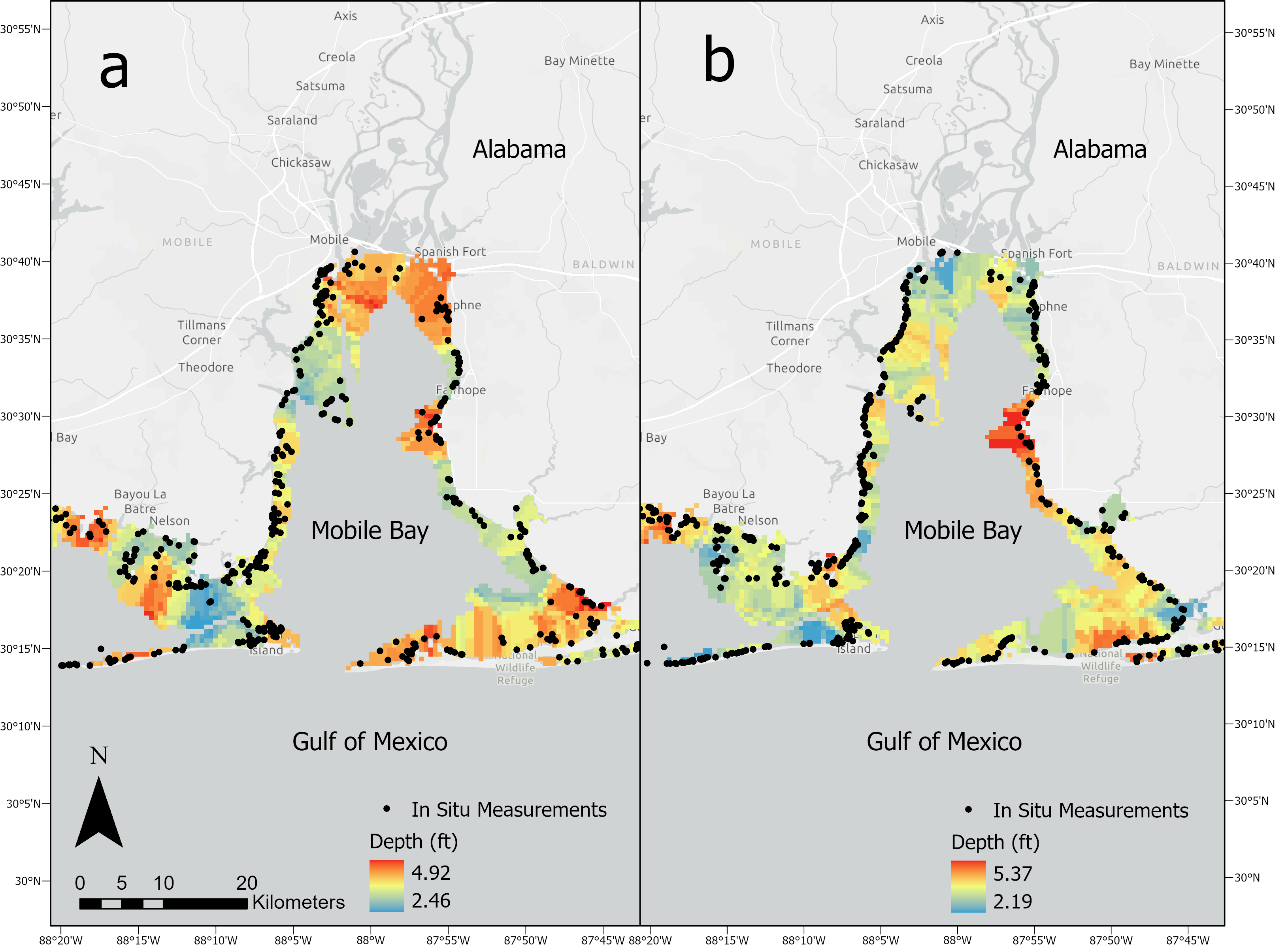

Supplement: Supplementary file 6 — Supplementary Figure S5. [file 41598_2024_54573_MOESM6_ESM.png]

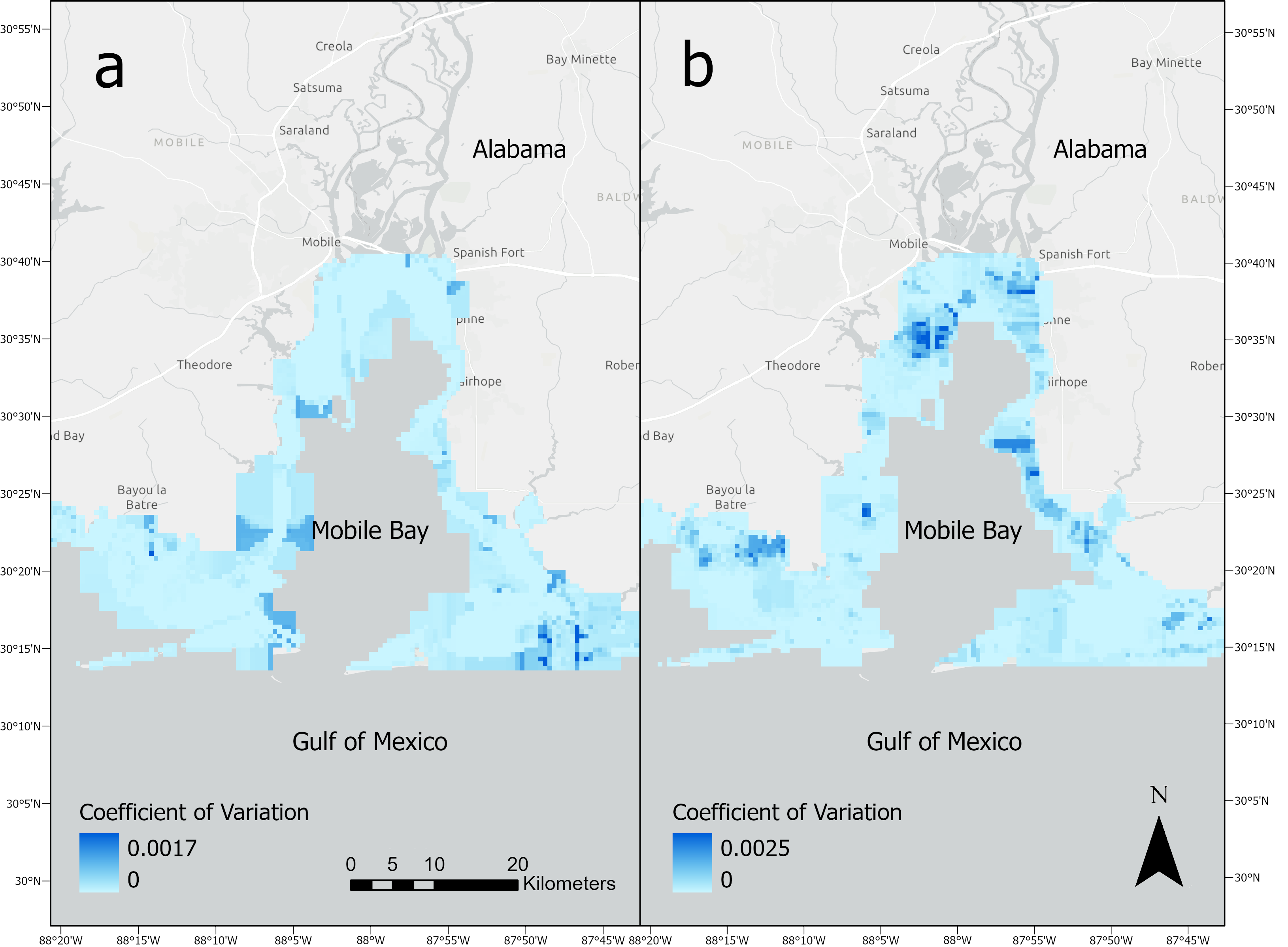

Supplement: Supplementary file 7 — Supplementary Figure S6. [file 41598_2024_54573_MOESM7_ESM.png]
